# Quality of palliative radiotherapy assessed using quality indicators: a multicenter survey^†^

**DOI:** 10.1093/jrr/rrae048

**Published:** 2024-06-26

**Authors:** Tetsuo Saito, Naoto Shikama, Takeo Takahashi, Naoki Nakamura, Takashi Mori, Kaori Nakajima, Masahiko Koizumi, Shuhei Sekii, Takeshi Ebara, Hiroki Kiyohara, Keiko Higuchi, Atsunori Yorozu, Takeshi Nishimura, Yasuo Ejima, Hideyuki Harada, Norio Araki, Misako Miwa, Kazunari Yamada, Terufumi Kawamoto, Nobuki Imano, Joichi Heianna, Miwako Nozaki, Yuki Wada, Yu Ohkubo, Nobue Uchida, Miho Watanabe, Takashi Kosugi, Kazunari Miyazawa, Shigeo Yasuda, Hiroshi Onishi

**Affiliations:** Division of Integrative Medical Oncology, Saiseikai Kumamoto Hospital, 5-3-1 Chikami, Minami-ku, Kumamoto-Shi, Kumamoto 861-4193, Japan; Department of Radiation Oncology, Juntendo University Graduate School of Medicine, 2-1-1 Hongo, Bunkyo-ku, Tokyo 113-8421, Japan; Department of Radiation Oncology, Saitama Medical Center, Saitama Medical University, 1981 Kamoda, Kawagoe-shi, Saitama 350-8550, Japan; Department of Radiation Oncology, St. Marianna University Hospital, 2-16-1 Sugao, Miyamae, Kawasaki-shi, Kanagawa 216-8511, Japan; Department of Radiation Oncology, Hokkaido University Hospital, Kita 14 Nishi 5, Kita-ku, Sapporo-shi, Hokkaido 060-8638, Japan; Department of Radiology, Asahikawa Medical University, 2-1 Midorigaoka-Higashi, Asahikawa-shi, Hokkaido 078-8510, Japan; Radiation Oncology Laboratory, Department of Medical Physics & Engineering, Graduate School of Medicine and Health Science, Osaka University, 1-7 Yamadaoka, Suita-shi, Osaka 565-0871, Japan; Department of Radiation Oncology, Kita-Harima Medical Center, 926-250 Ichibacho, Ono-shi, Hyogo 675-1392, Japan; Department of Radiation Oncology, Kyorin University, School of Medicine, 6-20-2 Shinkawa Mitaka-shi, Tokyo 181-8611, Japan; Department of Radiation Oncology, Japanese Red Cross Maebashi Hospital, 389-1 Asakura-Machi, Maebashi-shi, Gunma 371-0811, Japan; Department of Radiation Oncology, Isesaki Municipal Hospital, 12-1 Tsunatorihon-machi, Isesaki-Shi, Gunma 372-0817, Japan; Department of Radiation Oncology, NHO Tokyo Medical Center, 2-5-1 Higashigaoka, Meguro-ku, Tokyo 152-8902, Japan; Department of Radiation Oncology, Japanese Red Cross Society Kyoto Daiichi Hospital, 15-749 Honmachi, Higashiyama-ku, Kyoto-Shi, Kyoto 605-0981, Japan; Department of Radiology, Dokkyo Medical University, 880 Kitakobayashi, Mibu-machi, Shimotsuga-gun, Tochigi 321-0293 Japan; Radiation and Proton Therapy Center, Shizuoka Cancer Center, 1007 Shimonagakubo, Nagaizumi-cho, Sunto-gun, Shizuoka 411-8777, Japan; Department of Radiation Oncology, NHO Kyoto Medical Center, 1-1 Mukaihata-cho, Fukakusa, Fushimi-ku, Kyoto-shi, Kyoto 612-8555, Japan; Department of Radiation Oncology, Sendai Kousei Hospital, 1-20 Sutsumidori, Amemiya, Aoba-ku, Sendai-shi, Miyagi 981-0914, Japan; Department of Radiation Oncology, Seirei Mikatahara General Hospital, 3453 Mikatahara, Chuo-ku, Hamamatsu-shi, Shizuoka 433-8558, Japan; Department of Radiation Oncology, Juntendo University Graduate School of Medicine, 2-1-1 Hongo, Bunkyo-ku, Tokyo 113-8421, Japan; Department of Radiation Oncology, Graduate School of Biomedical Health Sciences, Hiroshima University, 1-2-3 Kasumi, Minami-ku, Hiroshima-shi, Hiroshima 734-8551, Japan; Department of Radiation Oncology, Nanbu Tokushukai Hospital, 171-1 Hokama, Yaese-cho, Shimajiri-gun, Okinawa 901-0493, Japan; Department of Radiation Oncology, Dokkyo Medical University Saitama Medical Center, 2-1-50 Minamikoshigaya, Koshigaya-shi, Saitama 343-8555, Japan; Department of Radiology, Akita University Graduate School of Medicine, 1-1-1 Hondo, Akita-shi, Akita 010-8543, Japan; Department of Radiation Oncology, Saku Central Hospital Advanced Care Center, 3400-28 Nakagomi, Saku-shi, Nagano 385-0051, Japan; Department of Radiation Oncology, Kawasaki Municipal Ida Hospital, 2-27-1 Ida, Nakahara-ku, Kawasaki,-shi, Kanagawa 211-0035, Japan; Diagnostic Radiology and Radiation Oncology, Graduate School of Medicine, Chiba University, 1-8-1, Inohana, Chuo-ku, Chiba-shi, Chiba 260-8670, Japan; Department of Radiation Oncology, Fujieda Municipal General Hospital, 4-1-11 Surugadai, Fujieda-shi, Shizuoka 426-8677, Japan; Department of Radiolgy, Showa General Hospital, 8-1-1 Hanakoganei, Kodaira-shi, Tokyo 187-8510, Japan; Department of Radiology, Chiba Rosai Hospital, 2-16 Tatsumidai-higashi, Ichihara-shi, Chiba 290-0003, Japan; Department of Radiology, University of Yamanashi, 1110 Shimokato, Chuo-shi, Yamanashi 409-3898, Japan

**Keywords:** quality indicator, evidence-practice gap, palliative radiotherapy, bone metastases, brain metastases, malignant spinal cord compression

## Abstract

We sought to identify potential evidence-practice gaps in palliative radiotherapy using quality indicators (QIs), previously developed using a modified Delphi method. Seven QIs were used to assess the quality of radiotherapy for bone metastases (BoM) and brain metastases (BrM). Compliance rate was calculated as the percentage of patients for whom recommended medical care was conducted. Random effects models were used to estimate the pooled compliance rates. Of the 39 invited radiation oncologists, 29 (74%) from 29 centers participated in the survey; 13 (45%) were academic and 16 (55%) were non-academic hospitals. For the QIs, except for BoM-4, the pooled compliance rates were higher than 80%; however, for at least some of the centers, the compliance rate was lower than these pooled rates. For BoM-4 regarding steroid use concurrent with radiotherapy for malignant spinal cord compression, the pooled compliance rate was as low as 32%. For BoM-1 regarding the choice of radiation schedule, the compliance rate was higher in academic hospitals than in non-academic hospitals (*P* = 0.021). For BrM-3 regarding the initiation of radiotherapy without delay, the compliance rate was lower in academic hospitals than in non-academic hospitals (*P* = 0.016). In conclusion, overall, compliance rates were high; however, for many QIs, practice remains to be improved in at least some centers. Steroids are infrequently used concurrently with radiotherapy for malignant spinal cord compression.

## INTRODUCTION

Clinical practice is not always performed in accordance with the guideline recommendations. Difficulties in implementing evidence-based practices have been demonstrated in palliative radiation oncology [1] and other areas of medicine [2, 3]. Quality indicators (QIs) are valuable tools for evaluating the quality of healthcare systems. Some QIs have been developed in radiation oncology [4]; however, surveys using them seem to be limited [5]. Previously, QIs have been developed to assess the quality of radiotherapy for bone metastases (BoM) and brain metastases (BrM) [6]. Additionally, these QIs were pilot tested in five centers, and the feasibility of their measurement was confirmed [6]. Radiotherapy for BoM and BrM is the standard of care for these diseases [7, 8]; however, its quality has scarcely been surveyed. To identify potential gaps between clinical practice and evidence in palliative radiotherapy, we conducted the present survey in radiation oncology centers in Japan.

## MATERIALS AND METHODS

Process QIs are widely used tools to evaluate the processes involved in health care [9]. Process QIs are presented as numerators and denominators (the percentage of patients for whom recommended medical care was conducted). In the present study, we used seven process QIs (Table 1), previously developed through a modified Delphi method [6], which is a method for determining expert consensus [10]. The QIs were developed through three online meetings and two e-mail surveys by a panel consisting of eight radiation oncologists, with expertise in palliative radiation oncology, and one expert on the Delphi methodology [6]. Of the seven QIs, four were on BoM and three were on BrM; the definitions of the denominators and numerators of the QIs are presented in Table 1. The denominator of the QI represents the number of patients for whom the QI was used to evaluate the quality of certain aspects of radiation oncology practice. The numerator represents the number of patients (among those in the denominator) for whom recommended care was provided. The compliance rate was calculated as the percentage of patients for whom practice was performed as recommended.

**Table 1:** Quality indicators

Quality indicators	Brief description	Definition of denominator	Definition of numerator	Total number of patients assessed	Pooled compliance rate (95% confidence interval)
BoM-1	Choice of radiation schedules	Patients who received radiation therapy for painful BoM^a^	Patients who received radiation therapy in ≤10 fractions, or for whom the reason for the use of extended-fractionation was written in the medical chart	435	99% (97–100%)
BoM-2	Assessment of pain before radiation therapy	Patients who received radiation therapy for painful BoM^a^	Patients for whom some description on pain before radiation therapy was written in the medical chart	435	97% (94–99%)
BoM-3	Prompt initiation of radiation therapy for clinical MSCC	Patients who received radiation therapy for clinical MSCC^b^	Patients for whom radiation therapy was initiated on the day of referral to radiation oncology or the next day	115	82% (68–93%)
BoM-4	Concurrent use of steroids with radiation therapy for clinical MSCC	Patients who received radiation therapy for clinical MSCC^b^	Patients for whom steroids were initiated or increased concurrently with the initiation of radiation therapy	115	32% (18–47%)
BrM-1	Assessment of performance status before radiation therapy	Patients who received radiation therapy for BrM	Patients for whom performance status before radiation therapy was recorded by radiation oncologists in the medical chart or radiology information system	288	92% (82–99%)
BrM-2	Completion of planned radiation therapy	Patients who received whole-brain radiation therapy for BrM	Patients for whom the planned radiation therapy was completed	215	97% (93–99%)
BrM-3	Initiation of radiation therapy without delay	Patients who received whole-brain radiation therapy for BrM^c^	Patients for whom the radiation therapy was initiated within 10 days from referral to radiation oncology	201	97% (92–99%)

BoM, bone metastases; BrM, brain metastases; MSCC, malignant spinal cord compression.

Patients with hematologic tumors should be excluded from the denominators in all the quality indicators.

^a^Patients who had received radiation therapy or surgery to the same bone metastases should be excluded from the denominator.

^b^When a symptom in the lower extremities, caused by spinal cord compression, was written in the medical chart or referral letter.

^c^Patients who received intensity modulated whole brain radiotherapy should be excluded from the denominator.

The present survey study was performed by members of the Japanese Society for Radiation Oncology (JASTRO) palliative radiotherapy committee and the Japanese Radiation Oncology Study Group (JROSG) palliative medicine committee. We evaluated the quality of palliative radiotherapy for BoM and BrM. For BoM, we evaluated painful BoM and clinical malignant spinal cord compression (MSCC). Patients with clinical MSCC were defined as those who were reported to have lower extremity symptoms caused by spinal cord compression in the medical chart or referral letter. Regarding BrM, we evaluated patients who received any radiation therapy for BrM and those who received whole-brain radiation therapy for BrM.

One panel member (N.S.) sent an e-mail to the members of the JASTRO palliative radiotherapy committee and the JROSG palliative medicine committee, inviting them to participate in a survey to evaluate the quality of palliative radiation oncology. Patients for whom the radiotherapy start date was between 1 January 2021 and 30 June 2021 were screened for eligibility for the study. The patients were screened consecutively from 1 January 2021. When the denominator of a QI reached 10, even if the screening period did not reach 30 June 2021, the screening of patients for the QI was allowed to be declared complete; full screening (i.e. from 1 January 2021 to 30 June 2021), while the denominator of the QI exceeded 10, was alternatively allowed. This study was approved by the Institutional Review Boards of Juntendo University (E22-0229), Seirei Mikatahara General Hospital (22-36), Shizuoka Cancer Center (T2022-40-2022-1-3), Nanbu Tokushukai Hospital (TGE02048-005), Fukuchiyama City Hospital (4-29) and Kyorin University (2039); the informed consent was waived.

Random effects models were used to estimate the pooled compliance rates. Compliance rates and 95% confidence intervals for each center were presented in a forest plot. Mixed effects models with *Q* tests were used to compare compliance rates between academic and non-academic centers. A *P*-value of <0.05 was considered statistically significant. Statistical analyses were performed using R version 4.2.2.

## RESULTS

Of the 39 invited members of the JASTRO palliative radiotherapy committee and the JROSG palliative medicine committee, 29 (74%) from 29 centers participated in the survey. Of the 29 centers, 13 (45%) were academic hospitals (12 university hospitals and one cancer center) and 16 (55%) were non-academic hospitals.

**Fig. 1:**
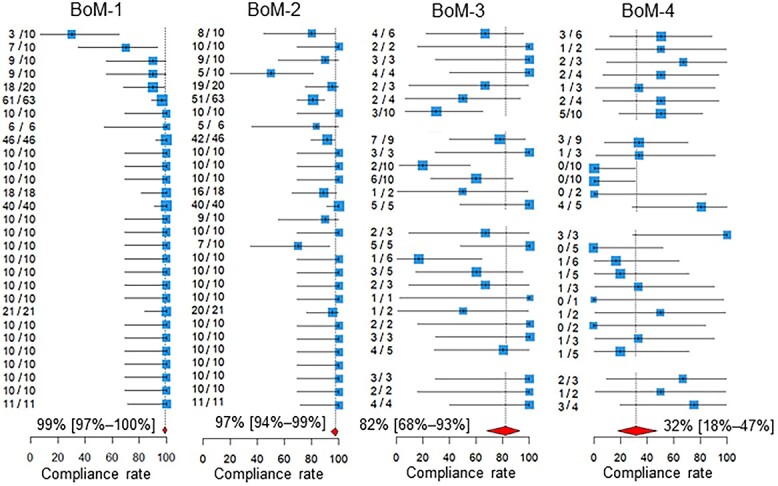
Compliance rates. Below the estimates and 95% confidence intervals of the compliance rates of the participating centers, the 95% confidence intervals of the pooled compliance rates are shown as diamonds. The two leftmost columns of numbers in each quality indicator are the numbers of patients for whom recommended medical care was performed and the total number of patients assessed in the participating hospitals. BoM, bone metastases; BrM, brain metastases.

**Fig. 2:**
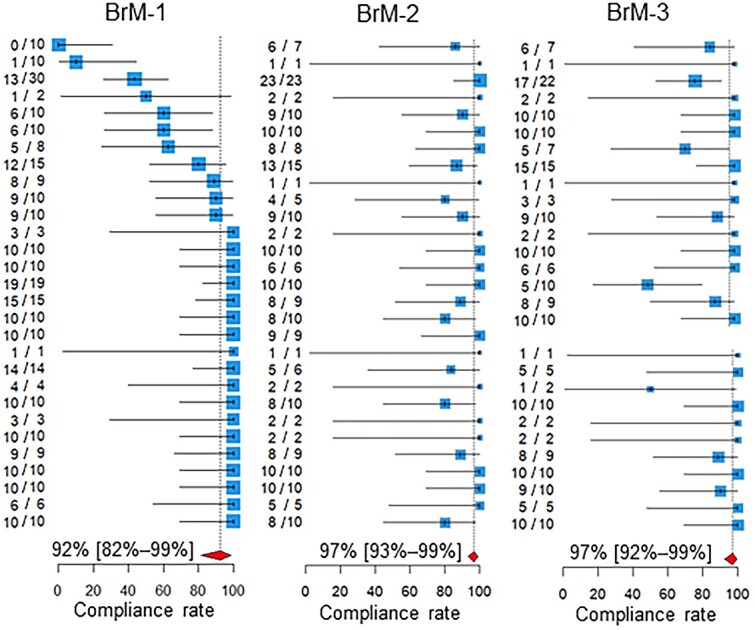
Compliance rates. Below the estimates and 95% confidence intervals of the compliance rates of the participating centers, the 95% confidence intervals of the pooled compliance rates are shown as diamonds. The two leftmost columns of numbers in each quality indicator are the numbers of patients for whom recommended medical care was performed and the total number of patients assessed in the participating hospitals. BoM, bone metastases; BrM, brain metastases.

The compliance rates are presented in Table 1 and Figs 1 and 2. In Fig. 1, the participating centers are shown in the order of magnitude of the estimates of the compliance rate for BoM-1; centers for which the denominator of the QI was zero are left blank. Similarly, in Fig. 2, the centers are shown in the order of the magnitude of the estimates of the compliance rate for BrM-1. The pooled compliance rates for all QIs were higher than 80%, except BoM-4, for which it was as low as 32%.

**Fig. 3:**
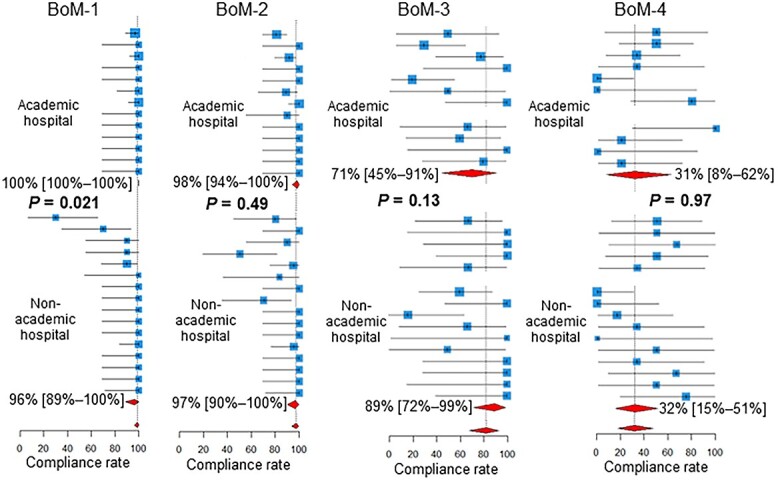
Compliance rates in academic vs non-academic centers. Below the estimates and 95% confidence intervals of the compliance rates of the participating centers, the 95% confidence intervals of the pooled compliance rates are shown as diamonds. BoM, bone metastases; BrM, brain metastases.

**Fig. 4:**
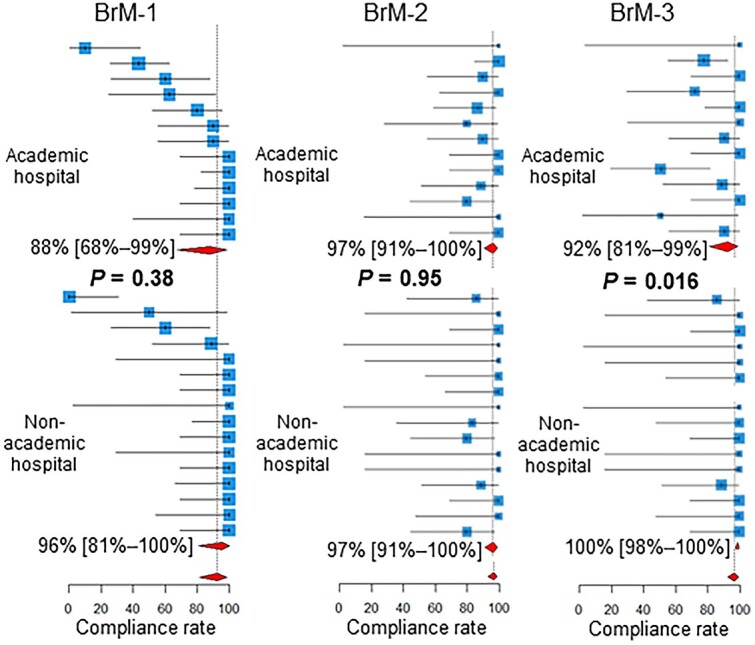
Compliance rates in academic vs non-academic centers. Below the estimates and 95% confidence intervals of the compliance rates of the participating centers, the 95% confidence intervals of the pooled compliance rates are shown as diamonds. BoM, bone metastases; BrM, brain metastases.

As shown in Fig. 3, for BoM-1, the compliance rate was higher in academic hospitals than in non-academic hospitals (*P* = 0.021). For the BrM-3, the compliance rate was lower in academic hospitals than in non-academic hospitals (*P* = 0.016, Fig. 4).

## DISCUSSION

The pooled compliance rates of all the QIs were higher than 80%, except BoM-4. One reason for these high compliance rates may be that we surveyed practices in centers where members of the JASTRO palliative radiotherapy committee and the JROSG palliative medicine committee worked. The quality of palliative radiation oncology at these centers may be higher than the average quality of radiation oncology centers in Japan. This potential bias in the selection of the centers surveyed highlights the finding that steroids are infrequently used concurrently with radiotherapy for MSCC.

The use of steroids concurrent with radiotherapy for clinical MSCC is widely recommended [11–16]. A randomized trial comparing high-dose dexamethasone and no dexamethasone concurrent with radiotherapy for MSCC found significantly higher ambulation rates in patients who received high-dose dexamethasone [17]. Small studies inconclusively indicated that high-dose steroids were not different from moderate-dose steroids in enhancing ambulation but were more frequently associated with serious adverse effects than were moderate-dose steroids [18–20]. Despite the lack of high-quality evidence to determine the appropriate dose of steroids, moderate-dose steroids (e.g. dexamethasone 16 mg per day) seem to be widely used for MSCC [21, 22].

Evidence exists suggesting that steroids should not be used routinely where a patient has good motor function [23]. In this single-arm trial [23], 20 patients with no neurologic deficits or only radiculopathy received radiotherapy for MSCC without steroids; the ambulation status was good after treatment in these patients with a median survival of 14 months. Therefore, we defined the BoM-4 denominator to include only patients with ‘clinical’ MSCC, excluding those with ‘radiological’ asymptomatic MSCC.

Our pooled compliance rate of 32% for the concurrent use of steroids with radiotherapy for clinical MSCC (BoM-4) was lower than those reported in previous single-center studies [24, 25]. In a retrospective audit of clinical practice on MSCC at a UK regional cancer center, in 42% of patients, dexamethasone had been prescribed before cancer center admission, and this increased to 96% following admission [24]. In a study based on the medical records of patients who received palliative radiotherapy for MSCC at a US university hospital, 80% and 88% of patients received steroids before and after the quality improvement initiative was introduced, respectively [25]. Although these studies have reported the utilization rate of steroids in single-center settings [24, 25], the present study may be the first multicenter survey to investigate the utilization rate of steroids concurrent with radiotherapy for MSCC.

Two previous studies that investigated the appropriate selection of dose schedules in radiotherapy for BoM [26, 27] reported comparable and lower compliance rates than ours (i.e. the pooled compliance rate of 99% for the use of fractions ≤10 for painful BoM [BoM-1]). In the Michigan Radiation Oncology Quality Consortium, among patients treated in the 28 participating radiation oncology practices, 97% and 98% of them who received radiotherapy for BoM received treatment with ≤10 fractions before and after the implementation of quality improvement measures (performed in 2019), respectively [26]. In another study based on the National Cancer Database, 60.2% of the patients with metastatic thoracic non-small cell lung cancer who were diagnosed between 2004 and 2016 and received radiotherapy for BoM received treatment with one of the following schedules: 30 Gy in 10 fractions, 24 Gy in 6 fractions, 20 Gy in 5 fractions or 8 Gy in a single fraction [27].

Compliance rates for the BoM-1 were higher in academic hospitals than in non-academic hospitals. Extended fractionation (≥11 fractions) for the BoM, which is not routinely recommended [27], appears to be performed less frequently in academic centers than in non-academic centers. In academic centers, practice may be more concordant with recommendations and guidelines than in non-academic centers. Another explanation is that the number of patients receiving radiotherapy tends to be higher in academic centers, and the burden of extended fractionation tends to be unacceptable in terms of machine time and manpower.

Compliance rates for the BrM-3 were lower in academic hospitals than in non-academic hospitals. Thus, the initiation of radiotherapy for BrM is more frequently delayed in academic centers than in non-academic centers. This may again reflect the heavier workload in academic centers.

This study had some limitations. First, there may have been bias in the selection of the centers surveyed. In the surveyed centers, the quality of palliative radiotherapy may have been, on average, high. Second, since the number of patients screened for eligibility for this study was not recorded, we could not evaluate how the assessed patients were selected from the patients who received palliative radiotherapy for BoM or BrM during the study period. Third, the primary sites of BoM and BrM were not recorded and, therefore, could not be analyzed. Fourth, the survey was conducted by physicians at their own facility rather than being audited by independent experts.

## CONCLUSION

In summary, we conducted a multicenter survey on the quality of palliative radiotherapy for BoM and BrM using previously developed and pilot-tested QIs. Overall, compliance rates were high; however, in many QIs, the practice remains to be improved in at least some centers. Despite supporting evidence and guideline recommendations, steroids seem to be underutilized concurrently with radiotherapy for MSCC. Approaches to improve the underutilization of steroids in the treatment of MSCC are required.

## CONFLICT OF INTEREST

N.S. was a member of the advisory board and has received honorariums from Elekta K.K.

## FUNDING

This study was supported by the Health Labor Sciences Research Grant from the Ministry of Health, Labor, and Welfare of Japan (JPMH21EA1010 and 23EA1012).

## References

[1] Ashworth A , Kong W, Chow E, et al. Fractionation of palliative radiation therapy for bone metastases in Ontario: do practice guidelines guide practice? Int J Radiat Oncol Biol Phys 2016;94:31–9. 26454681 10.1016/j.ijrobp.2015.07.2291

[2] Lomas J , Anderson GM, Domnick-Pierre K, et al. Do practice guidelines guide practice? The effect of a consensus statement on the practice of physicians. N Engl J Med 1989;321:1306–11. 2677732 10.1056/NEJM198911093211906

[3] De Schreye R , Smets T, Annemans L, et al. Applying quality indicators for administrative databases to evaluate end-of-life care for cancer patients in Belgium. Health Aff 2017;36:1234–43. 10.1377/hlthaff.2017.0199 28679810

[4] Harden SV , Chiew K-L, Millar J, et al. Quality indicators for radiation oncology. J Med Imaging Radiat Oncol 2022;66:249–57. 35243788 10.1111/1754-9485.13373 PMC9310822

[5] Mizuno N , Okamoto H, Minemura T, et al. Establishing quality indicators to comprehensively assess quality assurance and patient safety in radiotherapy and their relationship with an institution’s background. Radiother Oncol 2023;179:109452. 36572282 10.1016/j.radonc.2022.109452

[6] Saito T , Shikama N, Takahashi T, et al. Quality indicators in palliative radiation oncology: development and pilot testing. Adv Radiat Oncol 2021;7:100856. 35146217 10.1016/j.adro.2021.100856 PMC8818916

[7] Lutz S , Balboni T, Jones J, et al. Palliative radiation therapy for bone metastases: update of an ASTRO Evidence-Based Guideline. Pract Radiat Oncol 2017;7:4–12. 27663933 10.1016/j.prro.2016.08.001

[8] Gondi V , Bauman G, Bradfield L, et al. Radiation therapy for brain metastases: an ASTRO Clinical Practice Guideline. Pract Radiat Oncol 2022;12:265–82. 35534352 10.1016/j.prro.2022.02.003

[9] Lorenz KA , Dy SM, Naeim A, et al. Quality measures for supportive cancer care: the Cancer Quality-ASSIST Project. J Pain Symptom Manag 2009;37:943–64. 10.1016/j.jpainsymman.2008.05.018 19359135

[10] Eubank BH , Mohtadi NG, Lafave MR, et al. Using the modified Delphi method to establish clinical consensus for the diagnosis and treatment of patients with rotator cuff pathology. BMC Med Res Methodol 2016;16:56. 27206853 10.1186/s12874-016-0165-8 PMC4875724

[11] Loblaw DA , Perry J, Chambers A, et al. Systematic review of the diagnosis and management of malignant extradural spinal cord compression: the Cancer Care Ontario Practice Guidelines Initiative‘s Neuro-Oncology Disease Site Group. J Clin Oncol 2005;23:2028–37. 15774794 10.1200/JCO.2005.00.067

[12] Cole JS , Patchell RA. Metastatic epidural spinal cord compression. Lancet Neurol 2008;7:459–66. 18420159 10.1016/S1474-4422(08)70089-9

[13] National Institute for Health and Care Excellence . Metastatic Spinal Cord Compression in Adults: Risk Assessment, Diagnosis and Management. 2008. https://www.nice.org.uk/guidance/cg75/resources/metastatic-spinal-cord-compression-in-adults-risk-assessment-diagnosis-and-management-pdf-975630102469 (24 May 2023, date last accessed). 31820892

[14] Loblaw DA , Mitera G, Ford M, et al. A 2011 updated systematic review and clinical practice guideline for the management of malignant extradural spinal cord compression. Int J Radiat Oncol Biol Phys 2012;84:312–7. 22420969 10.1016/j.ijrobp.2012.01.014

[15] L’espérance S , Vincent F, Gaudreault M, et al. Treatment of metastatic spinal cord compression: cepo review and clinical recommendations. Curr Oncol 2012;19:e478–90. 23300371 10.3747/co.19.1128 PMC3503678

[16] Grávalos C , Rodríguez C, Sabino A, et al. SEOM clinical guideline for bone metastases from solid tumours (2016). Clin Transl Oncol 2016;18:1243–53. 27896639 10.1007/s12094-016-1590-1 PMC5138247

[17] Sørensen S , Helweg-Larsen S, Mouridsen H, et al. Effect of high-dose dexamethasone in carcinomatous metastatic spinal cord compression treated with radiotherapy: a randomised trial. Eur J Cancer 1994;30A:22–7. 8142159 10.1016/s0959-8049(05)80011-5

[18] Vecht CJ , Haaxma-Reiche H, van Putten WL, et al. Initial bolus of conventional versus high-dose dexamethasone in metastatic spinal cord compression. Neurology 1989;39:1255–7. 2771077 10.1212/wnl.39.9.1255

[19] Heimdal K , Hirschberg H, Slettebø H, et al. High incidence of serious side effects of high-dose dexamethasone treatment in patients with epidural spinal cord compression. J Neurooncol 1992;12:141–4. 1560260 10.1007/BF00172664

[20] Graham PH , Capp A, Delaney G, et al. A pilot randomised comparison of dexamethasone 96 mg vs 16 mg per day for malignant spinal-cord compression treated by radiotherapy: TROG 01.05 Superdex study. Clin Oncol 2006;18:70–6. 10.1016/j.clon.2005.08.015 16477923

[21] George R , Jeba J, Ramkumar G, et al. Interventions for the treatment of metastatic extradural spinal cord compression in adults. Cochrane Database Syst Rev 2015;2018:CD006716. 10.1002/14651858.CD006716.pub3. PMC6513178 26337716

[22] Kim KN , LaRiviere M, Macduffie E, et al. Use of glucocorticoids in patients with cancer: potential benefits, harms, and practical considerations for clinical practice. Pract Radiat Oncol 2023;13:28–40. 35917896 10.1016/j.prro.2022.07.003

[23] Maranzano E , Latini P, Beneventi S, et al. Radiotherapy without steroids in selected metastatic spinal cord compression patients. A phase II trial. Am J Clin Oncol 1996;19:179–83. 8610645 10.1097/00000421-199604000-00018

[24] McLinton A , Hutchison C. Malignant spinal cord compression: a retrospective audit of clinical practice at a UK regional cancer Centre. Br J Cancer 2006;94:486–91. 16434993 10.1038/sj.bjc.6602957 PMC2361169

[25] Mattes MD , Nieto JD. Quality improvement initiative to enhance multidisciplinary management of malignant extradural spinal cord compression. JCO Oncol Pract 2020;16:e829–34. 32384016 10.1200/JOP.19.00593 PMC7587429

[26] Jagsi R , Schipper M, Mietzel M, et al. The Michigan radiation oncology quality consortium: a novel initiative to improve the quality of radiation oncology care. Int J Radiat Oncol Biol Phys 2022;113:257–65. 35124133 10.1016/j.ijrobp.2022.01.048

[27] Grant SR , Smith BD, Colbert LE, et al. National quality measure compliance for palliative bone radiation among patients with metastatic non-small cell lung cancer. J Natl Compr Cancer Netw 2021;19:111–6. 10.6004/jnccn.2020.7688 34044365

